# Mitochondrial ROS Formation in the Pathogenesis of Diabetic Cardiomyopathy

**DOI:** 10.3389/fcvm.2020.00012

**Published:** 2020-02-18

**Authors:** Nina Kaludercic, Fabio Di Lisa

**Affiliations:** ^1^Neuroscience Institute, National Research Council of Italy (CNR), Padua, Italy; ^2^Department of Biomedical Sciences, University of Padua, Padua, Italy

**Keywords:** diabetic cardiomyopathy, reactive oxygen species, mitochondria, oxidative stress, diabetic complication

## Abstract

Diabetic cardiomyopathy is a result of diabetes-induced changes in the structure and function of the heart. Hyperglycemia affects multiple pathways in the diabetic heart, but excessive reactive oxygen species (ROS) generation and oxidative stress represent common denominators associated with adverse tissue remodeling. Indeed, key processes underlying cardiac remodeling in diabetes are redox sensitive, including inflammation, organelle dysfunction, alteration in ion homeostasis, cardiomyocyte hypertrophy, apoptosis, fibrosis, and contractile dysfunction. Extensive experimental evidence supports the involvement of mitochondrial ROS formation in the alterations characterizing the diabetic heart. In this review we will outline the central role of mitochondrial ROS and alterations in the redox status contributing to the development of diabetic cardiomyopathy. We will discuss the role of different sources of ROS involved in this process, with a specific emphasis on mitochondrial ROS producing enzymes within cardiomyocytes. Finally, the therapeutic potential of pharmacological inhibitors of ROS sources within the mitochondria will be discussed.

## Introduction

Chronic hyperglycemia, the major characteristic of type 1 diabetes (T1D), is a life-threatening risk factor that results in organ and tissue damage in the long term. One of the acute metabolic complications associated with mortality includes diabetic ketoacidosis occurring mainly in T1D (1). Instead, type 2 diabetes (T2D) and obesity are characterized by insulin resistance, hyperlipidemia and hyperinsulinemia that might occur before the onset of hyperglycemia. The heart is an insulin-dependent tissue, since insulin promotes glucose utilization and suppresses fatty acid utilization thereby conferring a certain level of metabolic flexibility, i.e., the ability to adapt substrate oxidation rates to substrate availability, in support of cardiac function (2). This metabolic flexibility is largely impaired in diabetic hearts, resulting in minimal glucose utilization, shift to free fatty acid utilization and energetic inefficiency (3). Vascular complications occurring in diabetes account for increased morbidity and mortality associated with this disease. In the long term, diabetes may cause microvascular disease, resulting from the damage of small blood vessels, and/or macrovascular disease, resulting from the damage of the arteries (4). The latter includes coronary artery disease, peripheral arterial disease, and stroke, while microvascular complications result in retinopathy, nephropathy and neuropathy. Diabetic cardiomyopathy (DCM) is a pathology associated with alterations in the myocardial structure and function without the coexistence of other cardiac risk factors such as coronary artery disease, hypertension, valvular disease (5). DCM is one of the deadliest complications associated with diabetes (1). The incidence of heart failure is increased in diabetic patients compared with age-matched individuals, independently of obesity, hypertension, dyslipidemia, and coronary artery disease (6). In addition, diabetes has also been associated with increased rates of cancer, physical and cognitive disability, tuberculosis and depression (7–12).

Reactive oxygen species (ROS) and oxidative stress have been linked both to the onset of diabetes and development of complications associated with this disease (13). Here, we will review the pathophysiological features of DCM, the evidence related to the contribution of ROS to DCM and the role of different sources of ROS involved in this process. The present review will focus on mitochondrial sources of ROS in cardiac myocytes (rather than other cell types in the heart) and will briefly discuss the advantages and disadvantages of targeting mitochondrial enzymes to prevent oxidant damage and postpone or prevent the development of cardiac complications in diabetes.

## Diabetic Cardiomyopathy

DCM is a result of diabetes-induced changes in the structure and function of the heart and is diagnosed only if there is cardiac dysfunction not associated with coronary artery disease (14). The clinical outcomes associated with ischemic heart disease, hypertension or heart failure are worse for patients with diabetes and indeed, cardiovascular complications are the leading cause of mortality and morbidity in diabetic patients (5, 15, 16). Thus, a better understanding of DCM-associated pathophysiology and underlying mechanisms is necessary in order to develop tools for early diagnosis and treatment strategies.

As an early complication of diabetes, DCM is characterized by a long latent phase during which the disease progresses, but is completely asymptomatic. This subclinical period includes an increase in the left ventricle (LV) mass, fibrosis, abnormalities in cell signaling and diastolic dysfunction (3, 5). Studies using magnetic resonance imaging demonstrated that hyperglycemia and insulin resistance are associated with an increase in LV mass (3, 17). The increase in cardiac stiffness and fibrosis detected in diabetic patients frequently evolves to heart failure with preserved ejection fraction (HFpEF) (18, 19). In some patients, diastolic dysfunction may progress to pump failure and impairment in systolic function resulting in heart failure with reduced ejection fraction (20, 21). Nevertheless, not all cardiac anomalies observed in T2D are recapitulated in T1D (22, 23). While T2D is characterized by both morphological and functional cardiac abnormalities in patients (i.e., LV hypertrophy, diastolic, and systolic dysfunction), T1D patients show intact systolic function and impairment in diastolic function (23). Moreover, not all studies conducted in T1D patients evidenced an impairment in diastolic function. This may be explained by the fact that T1D patients are normally treated with insulin that normalizes insulin-dependent metabolic processes and therefore likely renders T1D-induced alterations in the heart less evident (23). Regardless of these differences, clinical trials showed that the prevalence of heart failure in diabetic patients ranges from 19 to 26% (24–27), while the mortality rate is 15–20% in diabetic patients with systolic dysfunction (21).

**Figure 1:**
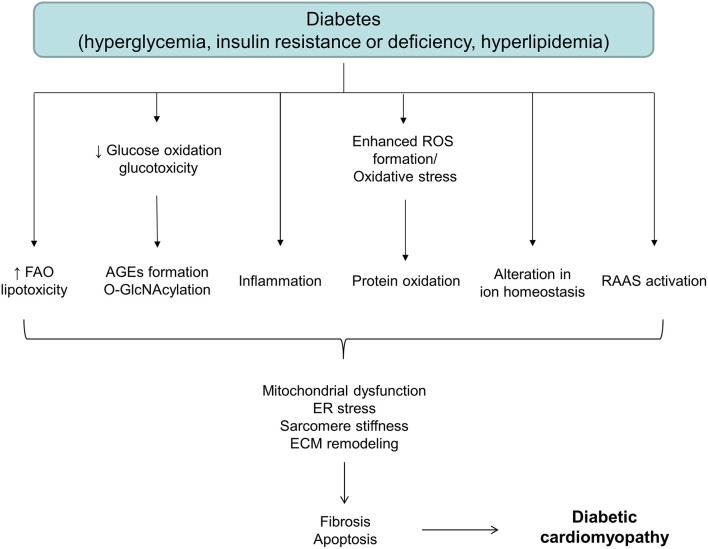
A schematic diagram depicting different factors involved in the onset and development of diabetic cardiomyopathy. AGEs, advanced glycation end products; ECM, extracellular matrix; ER, endoplasmic reticulum; FAO, fatty acid oxidation; RAAS, renin-angiotensin-aldosterone system; ROS, reactive oxygen species.

Although the exact mechanism of diabetes-associated LV dysfunction is not known, it appears that hyperglycemia, hyperinsulinemia, and/or lipotoxicity initiate a series of adaptive and maladaptive processes contributing to the development of heart failure. Factors underlying pathological changes in the diabetic heart are multiple. Metabolic alterations such as hyperglycemia, insulin resistance and increased free fatty acid levels, result in the oxidative stress, organelle dysfunction, inflammation, advanced glycation end products (AGEs) formation, activation of protein kinase C (PKC), abnormalities in ion homeostasis, alterations in structural proteins, apoptosis and fibrosis, changes that eventually result in diabetes-induced cardiac dysfunction (Figure 1) (28, 29). Despite a myriad of factors has been shown to collectively contribute to the development and progression of DCM, causal relationships and the exact sequence of events among these cellular and molecular mechanisms are still not entirely clear. Moreover, these factors frequently interact with each other, making DCM a very complex disease to treat.

## ROS: A Common Denominator in Diabetes-Induced Complications

ROS formation has gained significant experimental and clinical evaluation amongst the various mechanisms proposed (13, 20, 30). Notably, the aforementioned pathogenic factors and changes either induce or result from oxidative stress. ROS can be dangerous for biological systems for their capacity to interact with numerous macromolecules, such as proteins, lipids and DNA. ROS-induced modification of DNA can be mutagenic, especially if DNA damage cannot be repaired (31). ROS may lead to DNA strand breakage and formation of 8-hydroxydeoxyguanosine, a prominent feature in diabetic hearts (32, 33). While protein oxidation can be reversible and serve for signaling purposes, oxidative stress may lead to protein carbonylation that cannot be reversed and results in toxic aggregate accumulation if carbonylated molecules are not promptly degraded (34, 35). Membrane lipids are rich in polyunsaturated fatty acids that can easily be oxidized by ROS, a process that is also involved in the generation of atherosclerotic plaques (36). Lipid oxidation results in excess formation of carbonyl compounds, such as prostanoids and aldehydes, toxic metabolites that can promote numerous pathologies (37). In addition to direct macromolecule targeting, high ROS formation can also decrease the antioxidant capacity of the diabetic myocardium, contributing thereby to oxidative stress and resulting myocardial damage. This concept is further supported by studies demonstrating that overexpression of antioxidant defense proteins, such as metallothionein or catalase, could prevent oxidative stress and maladaptive remodeling of the diabetic hearts (22, 38–41). Mitochondrial ROS production underlies several hyperglycemia-induced pathogenic mechanisms, such as GAPDH inhibition, activation of polyol pathway, formation of AGEs, activation of PKC, glucose auto-oxidation, and activation of the 12/15-lipoxygenases pathway (13, 30, 42, 43). Activation of these pathways can, in turn, exacerbate oxidative stress. For instance, the polyol pathway utilizes NAPDH which is required for GSH regeneration, while binding of AGEs to their receptor results in ROS formation (44). Inhibition of AGE formation or AGE receptor gene knock-down attenuates the development of DCM (45). Moreover, activation of p53 signaling in T1D and T2D mouse models by an initial oxidative trigger leads to the upregulation of cytochrome c oxidase assembly protein, mitochondrial respiration, fatty acid oxidation, and mitochondrial ROS generation (46). However, hyperglycemia is not the only factor responsible for cardiac complications in diabetes, as mentioned before. Lipotoxicity and increased oxidation of free fatty acids also lead to oxidative stress, mitochondrial and ER stress, and activation of pro-inflammatory signals (47–51). Damage to mitochondria results in enhanced ROS generation and activation of the NLRP3 inflammasome (52) which, in turn, may promote or exacerbate cardiac fibrosis. Moreover, high glucose and inflammation provide a synergistic effect and further enhance ROS formation and all the downstream events leading to cell dysfunction (53, 54). Inflammation, angiogenesis, cardiomyocyte hypertrophy and apoptosis, fibrosis and contractile dysfunction, are processes susceptible to ROS-dependent modulation in the diabetic heart (55). Diastolic abnormalities observed in HFpEF are largely due to increased collagen and cardiomyocyte stiffness (56). ROS are well-known to target sarcomere proteins thereby inducing oxidative changes that may impact on sarcomere and cardiomyocyte stiffness (57, 58). While oxidation of the proteins forming the thick and thin filaments is mostly associated with impaired contractility, post-translational modifications of the elastic filament protein titin are tightly related to changes in LV stiffness (59). The passive stiffness of cardiac muscle was shown to be redox-dependent through titin oxidation and disulfide bridge formation that lead to increased cardiac stiffness (60). In addition to direct mechanisms, ROS can modulate sarcomere function also by affecting key protein kinases or phosphatases to induce post-translational modifications (57). In that regard, reduced titin phosphorylation is an important determinant of diastolic stiffness in HFpEF (59, 61, 62). This is particularly relevant in diabetic and obese patients in which oxidative stress impairs NO/cGMP/PKG signaling and leads to titin hypophosphorylation (59, 63) and increased cardiomyocyte stiffness along with collagen and AGEs deposition (59). Thus, enhanced ROS formation and alteration in the redox status are deeply intertwined with numerous alterations observed in diabetic hearts, suggesting that targeting ROS formation/elimination may represent an attractive therapeutic strategy for the treatment of DCM. Several cellular and subcellular sources that may account for enhanced ROS production were described in diabetic cardiovascular system and other tissues. Enzymes involved in deleterious ROS generation associated with diabetic complications include nicotinamide adenine dinucleotide phosphate oxidases (NOXs) (64–66), xanthine oxidase/oxidoreductase (XO) (67, 68), arachidonic acid cascade and microsomal enzymes, uncoupled nitric oxide synthase (NOS) (69), and mitochondria (13, 70–72).

NOX is a family of membrane-bound enzyme complexes composed of plasma membrane spanning and cytosolic components (73, 74). The active NOX complex allows for the transfer of electrons to molecular oxygen to generate superoxide (75). NOXs are considered to be one of the major cellular ROS sources and prominent players in several pathological conditions (74, 76, 77). NOX2, located in the cell membrane, and NOX4, localized in perinuclear ER and/or mitochondria, are expressed in the heart (78, 79). Increased cardiac NOX2 expression/activity has been described both in T1D and T2D, and contributes to hyperglycemia-induced ROS production (64–66, 80). NOX4 expression and NOX4-derived ROS are increased ~14 days after the induction of T1D in rats and contribute to the development of cardiomyopathy (81). Importantly, reducing either NOX2 or NOX4 activity in streptozotocin-induced diabetic hearts blunts myocardial oxidative stress, remodeling and improves cardiac function (81–83). ROS formation through NOX following high glucose administration has been associated with pathways involving sodium/glucose co-transporter 1 (SGLT1), PKCβ, and calcium/calmodulin dependent kinase II (CaMKII) (84). SGLT1-mediated glucose transport is responsible for NOX2 activation, since its inhibition efficiently abolished ROS production induced by exposure to high glucose (85). Importantly, PKCβ activation by RhoA/Rho kinase pathway activates Rac1 that, in turn, determines p47^phox^ translocation to the membrane, event required for NOX2 activation (86). Indeed, PKCβ inhibition by ruboxistaurin prevented NOX2 activation and subsequent ROS formation in cardiomyocytes treated with high glucose (87). An additional mechanism responsible for NOX activation in hyperglycemic conditions involves CaMKII activation. High glucose causes an increase in intracellular levels of Ca^2+^ that leads to CaMKII hyperphosphorylation and activation (32). Activated CaMKII is likely responsible for activation of PKCβ and downstream cascade of events (86). In that regard, inhibition of CaMKII prevented both the upregulation of p47^phox^ and p67^phox^ as well as oxidative stress in streptozotocin-induced model of T1D (32), suggesting that CaMKII may indeed play a major role in NOX-induced ROS formation.

XO is a cytoplasmic enzyme that catalyzes the oxidation of hypoxanthine to xanthine and further converts xanthine to uric acid (88). It uses oxygen as electron acceptor and produces superoxide and hydrogen peroxide (H_2_O_2_). In addition to their role in cardiac damage induced by ischemia/reperfusion injury or pacing-induced heart failure in dogs (89), hypoxanthine and XO activity are also increased in diabetic subjects (90). The role of XO in hyperglycemia-induced oxidative stress is documented by increased ROS formation in the muscle and development of fibrosis of hyperglycemic streptozotocin-induced diabetic mice (68, 91, 92). Some investigators reported evidence for beneficial vascular effects of XO inhibitors in hypercholesterolemic and diabetic patients (72, 93). Indeed, in T1D patients XO inhibition reduced the degree of oxidative stress, whereas in T2D patients it led to significant improvements in peripheral endothelium-dependent vasorelaxation (67, 90, 93).

NOS uncoupling results in superoxide formation, oxidative stress and decreased NO bioavailability that may have important vascular effects in diabetic subjects (94). Indeed, a decrease in the dimer to monomer ratio, indicative of the enzyme uncoupling, has been reported within the myocardium of diabetic animals (95). Consequently, inhibition of NOS activity and uncoupling by L-NAME, insulin-like growth factor, sepiapterin, ascorbic acid or N-acetyl-cysteine improved LV function in the diabetic heart (66, 96–100). In addition to uncoupling, NOS expression may also be increased in the diabetic hearts (33, 64, 101) and this is associated with an increase in lipid peroxidation and peroxynitrite generation (72). Peroxynitrite in turn may also lead to NOS uncoupling (102). Taken together, these studies suggest that the increased production of superoxide and peroxynitrite through NOS uncoupling is a major contributor to suppressed contractile performance in diabetes (72, 99, 100).

For detailed discussion related to XO, NOX or uncoupled NOS involvement in DCM, readers are referred to other excellent reviews (67, 72, 74, 84).

## Mitochondrial ROS Formation in DCM

The role of mitochondrial ROS formation and dysfunction in the pathogenesis of diabetes and its complications is well-established (13, 20, 28). Indeed, cardiac mitochondria from diabetic patients are dysfunctional, displaying increased mitochondrial H_2_O_2_ emission, impaired mitochondrial respiratory capacity and increased levels of oxidized or hydroxynonenal-modified proteins (103–105). Several mechanisms are likely responsible for mitochondrial dysfunction in diabetic hearts, including fatty acid-induced mitochondrial uncoupling, changes in mitochondrial morphology, increased ROS formation, mitochondrial proteome remodeling, impaired mitochondrial calcium handling and altered mitochondrial turnover (20, 28, 106–108). All these events might lead to compromised cardiac ATP generation and ultimately to cardiac dysfunction. Impairment in the activity of ATP synthase also affects mitochondrial function in the diabetic heart. A recent study very elegantly showed that hyperglycemia-induced calpain-1 upregulation in the mitochondria cleaves the ATP synthase α subunit, resulting in the reduction in the ATP synthase activity and increased mitochondrial ROS formation (109) that eventually contribute to the development of DCM. In addition, excessive mitochondrial ROS formation results in the increased propensity to permeability transition pore (PTP) opening that eventually leads to cell death (110). A tight relationship also exists between alterations in mitochondrial morphology and ROS formation that may reciprocally modulate each other. Cardiomyocytes from animal models of T1D, T2D, and from diabetic patients show increased levels of ROS and altered mitochondrial morphology, including mitochondrial fragmentation, cristae disruption and swelling (107, 108). Of interest, mitochondrial fragmentation induced by chronic hyperglycemia can be reversed with antioxidants, suggesting that ROS are causally related to this pro-fission phenotype and that controlling mitochondrial morphology and dynamics might represent a therapeutic strategy for the treatment of DCM (107, 111). Altered mitochondrial function may inhibit insulin signaling by interfering with oxidation of fatty acyl-CoA, accumulation of intracellular lipid and diacylglycerol, PKC activation and through generation of ROS (112). Both processes lead to insulin receptor substrate 1 phosphorylation and interference with insulin signal transduction. Reduction in mitochondrial ROS formation obtained either through cardiac-specific Mn-SOD overexpression or following stimulation of AMPK activity, prevented mitochondrial damage and many fatty acid- or hyperglycemia-induced events, both *in vitro* and *in vivo* (113–116).

**Figure 2:**
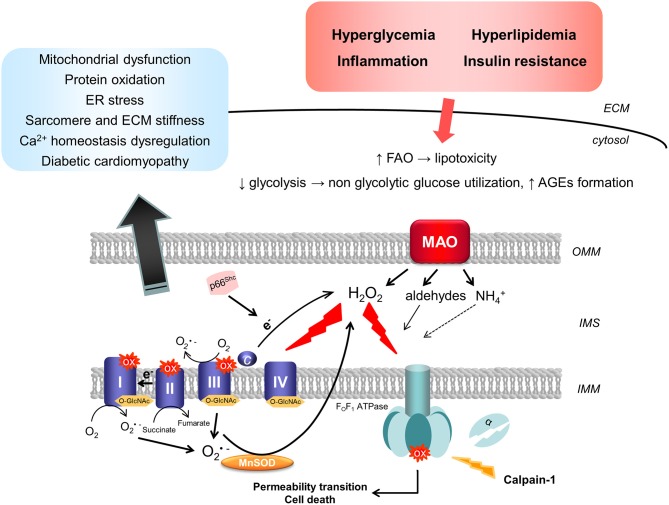
Mitochondrial sources of ROS in diabetic cardiomyopathy. Diabetic milieu, characterized by hyperglycemia, hyperlipidemia, and inflammation, results in the up-regulation in the activity of mitochondrial ROS-producing enzymes. Superoxide can be produced by the respiratory chain through forward or reverse electron transport. In addition, calpain-1 translocates to the mitochondrial matrix in the diabetic heart and cleaves the α subunit of the ATP synthase, leading therefore to the reduction in its activity and mitochondrial dysfunction. On the other hand, in situations of stress, p66^Shc^ is phosphorylated and translocates to the IMS where it catalyzes the electron transfer from cytochrome c to oxygen (O_2_) leading to the formation of hydrogen peroxide (H_2_O_2_). Finally, up-regulation of MAO activity upon exposure to high glucose and pro-inflammatory stimuli results in enhanced formation of H_2_O_2_ that can directly increase the susceptibility of mitochondria to undergo permeability transition. Post-translational modifications, such as oxidation or O-GlcNAcylation of respiratory chain complexes, can impair mitochondrial bioenergetics and function. All these events are implicated in the pathogenesis of diabetic cardiomyopathy by promoting mitochondrial and ER stress, leading to protein oxidation and Ca^2+^ homeostasis impairment, as well as sarcomere and ECM stiffness. AGEs, advanced glycation end products; ECM, extracellular matrix; ER, endoplasmic reticulum; FAO, fatty acid oxidation; IMM, inner mitochondrial membrane; IMS, intermembrane space; MAO, monoamine oxidase; MnSOD, manganese superoxide dismutase; OMM, outer mitochondrial membrane; O-GlcNAc, β-linked N-acetylglucosamine; ox, oxidation.

Given the tight relationship between mitochondrial ROS formation, structure/function, and diabetes-induced complications, it is crucial to dissect and identify sites responsible for ROS formation in mitochondria exposed to diabetic milieu. Electron transport chain (ETC), p66^Shc^, and monoamine oxidase (MAO) are the major sources of ROS formation in mitochondria (Figure 2).

### Electron Transport Chain

ETC is by far the major site of ATP production in mitochondria inside any given cell, and especially in cardiomyocytes (more than 90%). At the inner mitochondrial membrane (IMM), electrons from NADH and FADH_2_ are transferred across the respiratory chain to oxygen, which is reduced to water at the level of complex IV (117). This process powers the movement of protons into the intermembrane space and generates a proton gradient that drives the synthesis of ATP by the ATP synthase. A small amount of electrons (about 0.1%) can leak from the ETC and cause superoxide formation due to the partial reduction of oxygen (118). Superoxide generation may occur under conditions that decrease the flow of electrons, particularly at the level of the first three complexes where flavins or quinones are able to act as single electron donors (117, 119, 120). Notably, ROS formation can also result from the reverse electron flow through complex I (121). A recent study supported this pathophysiological concept demonstrating that succinate accumulates during cardiac ischemia *in vivo* (121, 122). Upon reperfusion, accumulated succinate is oxidized by complex II leading to dramatic ROS formation that is likely attributable to the reverse electron flow through complex I (122).

Seminal discoveries implicating ETC superoxide production as the central event in hyperglycemia-induced pathogenic mechanisms were provided by Brownlee's group back in 2000 using endothelial cells (123, 124). High intracellular glucose levels and glucose-derived pyruvate promote mitochondrial respiration by increasing the availability of reducing equivalents for the ETC and resulting in mitochondrial membrane hyperpolarization and superoxide production (123, 125). Furthermore, hyperglycemia-induced ROS formation is prevented by several interventions, such as via inhibition of ETC complex II activity, uncoupling of oxidative phosphorylation, by overexpression of uncoupling protein-1 and/or Mn-SOD (123). Normalizing levels of mitochondrial ROS with each of these agents prevents glucose-induced activation of PKC, hexosamine pathway, formation of AGEs, sorbitol accumulation, and NFκB activation. A further confirmation that ETC superoxide production is responsible for these events comes from experiments performed in Rho zero (ρ0) endothelial cells lacking mitochondrial ETC function (30). When exposed to high glucose, ρ0 cells do not display an increase in ROS production. Similar mechanism has also been shown to be at play in cardiomyocytes exposed to high glucose. Indeed, ROS formation is reduced in cardiomyocytes isolated from diabetic animals in which complex I and II activity is inhibited or which overexpress catalase, further denoting the crucial role of ETC in ROS generation in diabetes (41, 86, 126). Interestingly, the protective effect afforded by complex I or II inhibition suggests that ETC superoxide production upon high glucose exposure likely occurs through the reverse electron transport. It remains to be elucidated whether succinate accumulation occurs at some point during the development of cardiovascular complications in diabetes. Cardiac lipotoxicity is also mediated by mitochondrial ROS formation. Indeed, exposure to palmitate enhances mitochondrial ROS generation and leads to increased mitochondrial fission by modulating DRP1 phosphorylation levels and proteolytic processing of OPA1 (47).

An initial ROS trigger produced by ETC can promote activation of processes that eventually amplify the signal and lead to oxidative stress. Such processes involve the occurrence of post-translational modifications, such as (but not limited to) diabetes–induced defects caused by oxidation, increased methylglyoxal adduct formation and increased O-GlcNAcylation, that contribute to the impairment in mitochondrial and systolic function (127–129). Hyperglycemia alters the function of respiratory chain in mitochondria via dysregulation of O-GlcNAcylation (130, 131). O-GlcNAc transferase (OGT) enzyme is located in the IMM and interacts with complex IV of the respiratory chain in normal conditions. In streptozotocin-treated rats this enzyme is improperly localized to the mitochondrial matrix and the impairment in the OGT-complex IV interaction results in the loss of complex IV activity and reduced mitochondrial membrane potential (130). O-GlcNAcylation of proteins involved in mitochondrial dynamics, such as DRP-1 and OPA1, also contributes to mitochondrial fragmentation that further exacerbates organelle dysfunction (132, 133). On the other hand, methylglyoxal-induced modifications affect Ca^2+^ homeostasis and indirectly affect mitochondrial function. Indeed, in the diabetic heart methylglyoxal preferentially forms adducts with proteins involved in the intracellular calcium handling such as ryanodine receptor 2 and SERCA2a (134, 135). Ryanodine receptor glycation is associated with impaired Ca^2+^ cycling, increased mitochondrial Ca^2+^ levels and mitochondrial dysfunction (136). Collectively, these studies underline the importance of ETC-derived superoxide in diabetic conditions and mitochondria as their source and target.

### p66^Shc^

p66^Shc^ is another important source of ROS in mitochondria. p66^Shc^ is a cytosolic adaptor protein and, along with p46^Shc^ and p52^Shc^, is encoded by the ShcA gene (137, 138). p46^Shc^ and p52^Shc^ isoforms are formed through alternative translation start sites (137, 139). While p46^Shc^ and p52^Shc^ isoforms are ubiquitously expressed, p66^Shc^ promoter may bear epigenetic modifications resulting in cell type- or specific condition-restricted expression (140). Under stress conditions, PKCβ phosphorylates p66^Shc^ at Ser-36, event required for its translocation to mitochondria (141). Once in the intermembrane space, p66^Shc^ catalyzes the electron transfer from cytochrome c to oxygen resulting in the formation of H_2_O_2_ (142). In addition to this mechanism, p66^Shc^ can promote oxidative stress by activating membrane-bound NOX or through down-regulation of antioxidant enzymes synthesis (143). Accordingly, cells and mice lacking p66^Shc^ show reduction in markers of oxidative stress (139, 144).

A number of studies characterized the pathophysiological role of p66^Shc^ in cardiovascular diseases, such as maladaptive hypertrophy, heart failure and ischemia/reperfusion injury (137–139, 145). Importantly, excessive ROS generation is a major contributing factor to those cardiac pathologies (146). Since PKC activation plays a major role in the intracellular signaling leading to oxidative stress, cell dysfunction and tissue damage in hyperglycemia, and is required for p66^Shc^ translocation to mitochondria in response to stress (70), it is tempting to hypothesize that p66^Shc^ may play a role in cardiovascular complications induced by hyperglycemia acting as a downstream target following high glucose-induced PKCβ activation. Indeed, p66^Shc−/−^ mice have an increased resistance to ROS (70), less atherosclerosis and preserved aortic endothelium-dependent vasorelaxation following high-fat diet and in a model of streptozotocin-induced T1D (147, 148). Moreover, lack of p66^Shc^ prevented oxidative damage in cardiac progenitor cells and cardiomyocytes in streptozotocin-induced DCM (149). Unlike diabetic wild type animals characterized by cardiomyocyte loss, diabetic p66^Shc−/−^ hearts displayed preserved cardiac progenitor cell replication and turnover, along with unaltered wall thickness, chamber volume, LV end-diastolic pressure and diastolic wall stress (149).

### Monoamine Oxidases

Monoamine oxidases (MAOs) are flavoenzymes localized at the level of the outer mitochondrial membrane. MAOs exist in two isoforms, A and B, differing in structure, substrate preference, inhibitor specificity and tissue distribution (150–153). The physiological role of MAOs consists in the catalysis of the oxidative deamination of its substrates (i.e., endogenous and exogenous amines, neurotransmitters). MAOs generate H_2_O_2_, ammonia and corresponding aldehydes as products of catalysis (154, 155). Over the last decade, several studies have shown that alterations in redox balance cause by enhanced MAO activity play a prominent role in promoting the development of cardiovascular disorders and causing oxidative damage to cardiomyocytes (37, 146, 156–158). Indeed, MAO contributes to ischemia/reperfusion injury, maladaptive hypertrophy, heart failure and vascular dysfunction (37, 139, 159–162). Of note, evidence for MAO involvement in cardiac disease has also been demonstrated in patients. Up-regulation of MAO activity and consequent ROS formation has been identified as a prominent contributor to the impaired myocardial redox balance in patients and a major risk factor and predictor for the postoperative atrial fibrillation (163). In addition, MAO activity was shown to be increased in left and right ventricles from patients with ischemic heart disease (164). With regard to the possible involvement of MAO in diabetes, one study showed an improvement in blood glucose levels and systolic and diastolic pressures in a patient with T1D administered with the MAO inhibitor tranylcypromine (165). Unexpectedly, it has been demonstrated that pioglitazione, used as an antidiabetic drug in T2D patients, is a specific and reversible MAO-B inhibitor (166). These findings support a possible MAO involvement in diabetes-induced complications.

A clear and undeniable evidence for the role of MAO in the pathogenesis and progression of DCM came from animal models of T1D showing that MAO inhibition prevents cardiac dysfunction, death and fibrosis in diabetic mice and rats (71, 167). Data from our laboratory indicates that MAO activity is responsible for diastolic stiffness and dysfunction, some of the earliest signs of DCM in diabetic mice (71). Indeed, administration of MAO inhibitors is able to prevent oxidative changes, diastolic dysfunction and myocardial fibrosis in streptozotocin-treated hearts. In addition, MAO inhibition prevented mast cell degranulation in diabetic hearts, event that can contribute to fibrotic remodeling of the myocardial tissue. This evidence suggests that MAO-generated ROS are at the basis of diabetes-induced cardiovascular complications and, in addition to cardiomyocytes, affect also other cell types present in the heart. Oxidative stress induced by enhanced MAO activity has also been implicated in cardiomyocyte and mesenchymal stromal cell senescence (168–170). It remains to be elucidated whether ROS produced by MAO may also promote cardiac progenitor cell senescence during remodeling induced by diabetes, as is the case with p66^Shc^.

Up to date, the mechanisms underlying MAO toxicity have mostly been attributed to excessive H_2_O_2_ and aldehyde formation that leads to impaired mitochondrial function (146). Our recent work showed that incubation of primary cardiomyocytes with high glucose and pro-inflammatory cytokine IL-1β leads to a MAO-dependent increase in ROS that, in addition to causing PTP opening and mitochondrial dysfunction, also results in the endoplasmic reticulum (ER) stress (71). This evidence indicates that, in addition to mitochondrial ROS being a trigger for inflammasome activation, inflammatory processes can also promote mitochondrial ROS formation by up-regulating MAO activity. MAO inhibition prevented mitochondrial dysfunction and ER stress, factors that eventually contribute to the progression of DCM, suggesting that cardiomyocyte targeting of pro-inflammatory stimuli occurs in a MAO-dependent manner (71). Given that MAO is localized at the outer mitochondrial membrane and faces the cytosol, it is conceivable to imagine that H_2_O_2_ produced by MAO can also affect the function of neighboring organelles. Notably, ER and mitochondria are adjacent organelles, connected both at structural and functional level (171). Although our data suggests that MAO-induced mitochondrial dysfunction occurs upstream of ER stress, it is tempting to hypothesize that MAO may directly modulate ER function also through physical interaction with ER-resident proteins (mitochondria associated membrane proteins, for instance). Finally, it cannot be excluded that other products of MAO activity, such as aldehydes, may also contribute to diabetes-induced alterations. MAO-dependent oxidative stress may lead to the inhibition of aldehyde dehydrogenase 2 (ALDH2) resulting in further accumulation of toxic and reactive aldehydes (37). In that regard, it has been demonstrated that stimulation of ALDH2 activity protects from streptozotocin-induced cardiac damage (172), suggesting that accumulation of aldehydes may promote cardiac remodeling in diabetes independently or in concert with high ROS levels (173).

## Feed-Forward/Amplification Loop for ROS Formation

An intense cross-talk between different cellular ROS sources is likely to exist since many papers report that inhibition of a single ROS source prevents the development of cardiac pathology triggered by oxidative stress (146). For instance, hyperglycemia does not induce ROS formation in the ρ0 cells in which the respiratory chain is disrupted, as well as upon NOX or MAO inhibition (30). In addition, mitochondrial superoxide scavenging using mitochondria-targeted antioxidants is able to reduce NOX2 expression and activity in diabetic myocardium (174), while genetic inhibition of NOX2 and consequent reduction in superoxide formation at the mitochondrial level suggest that mitochondrial ROS formation in hyperglycemic hearts might be NOX2-dependent (82–84). Such evidence strongly supports the existence of an “amplification mechanism,” whereby an initial stress (i.e., hyperglycemia and/or inflammation), induces the formation of ROS that, in turn, activates other ROS producing enzymes to start producing free radicals thus amplifying the original oxidative trigger (146). The hypothesis of the feed-forward/amplification mechanism is also supported by the characterization of the so-called ROS-induced ROS release mechanism, whereby an initial ROS trigger induces PTP opening that leads to further ROS formation, instituting thereby a positive feedback loop for the ROS-induced ROS release (175, 176). This is indeed the case in adult cardiomyocytes that, when exposed to high glucose and pro-inflammatory stimuli, display an increase in MAO-dependent ROS formation that causes PTP opening and mitochondrial and ER stress (71). Moreover, other processes may participate in such amplification loop, such as for instance impairment in autophagy. While low/moderate ROS levels are required for autophagy initiation, excessive oxidative damage can impair autophagy resulting in the aberrant clearance of damaged proteins and/or organelles (177–180). For instance, AGE accumulation in an experimental model of diabetes inhibits autophagy, induces ER stress and promotes ROS formation (181). Either autophagy stimulation with rapamycin or inhibition of ER stress due to ER chaperone administration alleviate AGEs-induced deleterious effects on cardiomyocytes, suggesting that these processes are involved in diabetes-induced cardiac remodeling. Impairment in the elimination of damaged and dysfunctional mitochondria in diabetic hearts results in the accumulation of ROS-producing fragmented mitochondria (182, 183). Either inhibition of mitochondrial fragmentation during exposure to high glucose or stimulation of organelle removal through mitophagy in high-fat diet fed animals prevents oxidative stress as well as mitochondrial and cardiac dysfunction (182, 184, 185). However, it appears that autophagy and mitophagy are independently controlled in T2D, since autophagy flux was attenuated following 6 weeks of high-fat diet while mitophagy continued to increase even after 2 months (184). This suggests that mitophagy may occur through a non-canonical, alternative autophagy pathway. In this regard, it has been previously shown that Rab9 is mobilized to the mitochondria in early stages of diabetes where it induces activation of alternative autophagy for mitophagy (186, 187). The mechanisms controlling the activation of canonical vs. non-canonical autophagy remain unknown to date. Mitochondrial ROS are implicated in the activation of the canonical autophagy (178), but on the other hand excessive mitochondrial ROS formation impairs lysosomal biogenesis, function and the autophagy process in the cardiac myocytes (156, 168). Whether alterations in the redox status may represent the switch for autophagy to become maladaptive, and/or for the activation of canonical vs. non-canonical autophagy remains to be defined.

## Interventions Aimed at Reducing ROS Burden in DCM

Given the large body of evidence linking aberrant ROS formation and oxidative stress to the development of cardiac diseases, it is quite straightforward to hypothesize that reducing redox burden would protect the heart against deleterious changes induced by diabetes or other pathologies. Nevertheless, large scale clinical trials using antioxidant therapies have not produced the desired results (188, 189). Whether this is a consequence of particular antioxidant molecules used in clinical trials, their limited absorption and/or reduced cardiac availability, or it can be explained by the fact that a certain level of ROS is beneficial and required for signaling and physiological processes, including the response to insulin mediated by p66^Shc^-dependent ROS (190), remains to be elucidated. Another attractive explanation is that interfering with the complex redox network might result in compensatory changes (191). Currently, there are no efficient therapies to treat HFpEF in patients with diabetes. In that regard, antidiabetic SGLT2 inhibitors (such as empagliflozin) afforded cardioprotective effects in patients with diabetes (192). SGLT2 inhibitors lead to the reduction in plasma volume and reduced preload, events that have a favorable effect on cardiac function and structure (193, 194). Importantly, human and rodent hearts do not express SGLT2 (195–197), suggesting that the direct cardioprotective effects of SGLT2 inhibitors are independent of their action on SGLT2. Indeed, it has been demonstrated that SGLT2 inhibitors can directly affect cardiomyocytes by targeting Na^+^/H^+^ exchanger 1, reducing intracellular Na^+^ and Ca^2+^ levels, improving mitochondrial function and reducing inflammation and AMPK activity (197, 198). In addition, SGLT2 inhibitors are able to reduce oxidative stress through Nrf2/ARE signaling activation and it is likely that these off-target effects contribute to the cardioprotection observed in clinical trials (198–200). Another strategy to modulate an oxidative stress-related pathway is the use of the soluble guanylate cyclase stimulator vericiguat that targets the cGMP pathway in an ROS/NO-independent manner (201). HFpEF is associated with excessive ROS formation by the coronary microvasculature that limits NO bioavailability, reduces cGMP levels and therefore lowers PKG activity (as discussed in section 3). A recent clinical trial demonstrated an improvement in quality of life in patients with HFpEF receiving vericiguat for 12 weeks, suggesting that it could be a promising therapeutic agent in HFpEF (201). To foster the development of a specific and successful therapy, future studies should aim either at identifying the molecular ROS targets (191), the pathways of redox signaling or the specific sources of ROS that are responsible for deleterious changes in the diseased heart. While the first two options are just beginning to become accessible and are still far from being conclusively elucidated, inhibition of specific ROS sources might prove to be a useful strategy to prevent alterations in the redox status, and myocardial structure and function.

In this regard, inhibitors for some of the ROS sources outlined in this review are being developed and/or tested in the clinic. Data obtained in experimental models of diabetes identified NOX4 as a therapeutic target (81). Indeed, NOX4 inhibitors are currently being tested for various cardiovascular indications (76, 202). For instance, GKT-831 is a NOX1/4 dual inhibitor and the only NOX inhibitor that has reached the clinical trial stage; in fact, it is currently being tested in clinical trial phase II for diabetic nephropathy. It remains to be established whether NOX inhibitors would be effective in limiting cardiovascular complications in diabetic patients.

In line with the concept of mitochondria as major ROS producers, employment of mitochondria-targeted antioxidants such as MitoTEMPO proved to be cardioprotective in experimental models of DCM (203). On the other hand, MitoQ was never tested in such setting and neither of the compounds was ever tested in clinical trials. The paucity of studies concerning the use of mitochondrial antioxidants in DCM urges for studies adopting strategies that target specific mitochondrial ROS sources or their downstream targets (204). In that regard, it is not possible to inhibit the respiratory chain in humans in the long term without jeopardizing a wide array of vital functions. Although genetic inhibition of p66^Shc^ has proven protective in many cardiovascular pathologies, pharmacological inhibitors of p66^Shc^ are not yet available. On the contrary, MAO inhibitors are clinically available and employed for the treatment of depression and neurodegenerative diseases (76, 164, 205–207). As mentioned before, administration of a non-selective MAO inhibitor to a patient with T1D led to several improvements, including those at the cardiovascular level (165). Side-effects associated with the “old” irreversible MAO-A inhibitors have been eliminated since reversible MAO-A inhibitors or selective MAO-B inhibitors have been developed (207). Taking into consideration recent findings obtained in experimental models of DCM, it is worth assessing whether molecules such as moclobemide or safinamide could be repurposed for the treatment of patients with DCM.

## Conclusions

Current consensus is that exacerbated ROS generation due to hyperglycemia and/or fatty acid oxidation causes oxidative stress, that in turn promotes the development and progression of diabetes and its complications. In addition to the cytosolic sources of ROS, it is now well-documented that mitochondrial sources represent the major ROS burden in multiple tissues in both animal and human diabetic subjects. Pharmacological targeting of specific ROS sources may prove as a successful therapeutic strategy for the treatment of DCM. Alternatively, identification of processes and targets downstream of mitochondrial ROS may hold more promise in correcting cellular structural and functional derangements in diabetic individuals.

## Author Contributions

All authors listed have made a substantial, direct and intellectual contribution to the work, and approved it for publication.

### Conflict of Interest

The authors declare that the research was conducted in the absence of any commercial or financial relationships that could be construed as a potential conflict of interest.
